# HSF/HSP Networks and Environmental Memory: From Molecular Persistence to a Recovery-Associated State

**DOI:** 10.3390/ijms27219890

**Published:** 2026-11-04

**Authors:** Monica De Caroli, Carla Perrotta, Patrizia Rampino

**Affiliations:** 1 Department of Biological and Environmental Sciences and Technologies, University of Salento, Via Monteroni 165, 73100 Lecce, Italy; monica.decaroli@unisalento.it (M.D.C.); carla.perrotta@unisalento.it (C.P.); 2 NBFC National Biodiversity Future Center, 90133 Palermo, Italy

**Keywords:** HSF/HSP network, environmental memory, thermomemory, stress priming, recovery-associated state, proteostasis, heat stress, environmental history

## Abstract

Plants encounter environmental stresses in sequences of exposure, recovery, and subsequent challenge. Persistent molecular changes alone do not establish how previous exposure influences later responsiveness. Sustained protection may contribute to memory, whereas unresolved physiological disturbance can complicate its interpretation. The heat shock factor/heat shock protein (HSF/HSP) system links transcriptional regulation with protein folding, quality control, and recovery, providing a framework for examining these distinctions. We assess evidence for HSF/HSP-dependent environmental memory through its temporal organisation. Recurrent heat provides the strongest causal evidence, implicating HSF activity, transcriptional and chromatin regulation, chaperone persistence, and protein turnover in distinct aspects of thermomemory. Broader stress histories reveal altered subsequent responses, but causal HSF/HSP involvement remains less clearly resolved. We define experimental criteria for characterising recovery, demonstrating history-dependent functional responses, and identifying when candidate processes contribute causally. We then propose a recovery-associated state as a heuristic working model, rather than a demonstrated discrete cellular entity, to examine how previous exposure changes the configuration of regulatory and proteostatic processes during recovery. Persistent protective components, altered relationships among processes, or consequences outlasting the initiating molecular change may shape the later response. This framework guides experiments across stress sequences and species while distinguishing established thermomemory mechanisms from hypotheses about broader environmental memory.

## 1. Introduction

Plants rarely experience environmental stress as isolated events. Fluctuations in temperature, water availability, irradiance, and other environmental variables generate histories in which the response to a current challenge can depend on previous exposure, the interval between stresses, and the extent of recovery [1,2]. The resulting phenotype may reflect several processes, including sustained protection, incomplete recovery, persistent metabolic or hormonal changes, altered transcriptional competence, chromatin modifications, and changes in protein homeostasis [1]. Determining how these processes contribute to subsequent responsiveness is essential for understanding environmental memory.

The heat shock factor/heat shock protein (HSF/HSP) system is particularly relevant to this problem because it integrates stress sensing, transcriptional regulation, protein folding, protein turnover, and recovery. Heat stress provides the clearest experimental framework. In *Arabidopsis*, HSA32, HSP21, HSFA2, HSFA3, FtsH6, and components of the transcriptional machinery have each been shown to contribute to distinct aspects of persistent heat responsiveness after an intervening recovery period [3,4,5,6,7]. These studies demonstrate that selected processes associated with HSF/HSP can make causal contributions to thermomemory, but they also show that the relevant molecular requirements differ in time and function.

Recent work further indicates that memory competence depends on properties of HSF proteins beyond their abundance, including protein architecture and higher-order organisation [8,9]. These findings reinforce the view that persistent stress responsiveness cannot be reduced to continued expression of a single memory-associated HSF or HSP.

This distinction becomes more important when environmental history extends beyond recurrent heat. Drought followed by heat, repeated drought, or sequential combinations of different stresses can alter subsequent responses, but such phenotypes are difficult to interpret because physiological effects of the first exposure may influence the response to the later challenge. An increase in HSF/HSP expression after a second stress, for example, may reflect altered stress severity or a history-dependent change in regulatory competence. Neither molecular persistence nor expression during rechallenge establishes which process mediates the effect of previous exposure.

Previous reviews have extensively synthesised research on plant stress memory and thermomemory, including the roles of HSFs and HSPs during heat stress [10,11,12]. However, the relationship between these areas remains less clearly resolved when environmental history extends beyond recurrent heat. In particular, it remains necessary to establish causal HSF/HSP contributions while accounting for incomplete recovery, and to determine how persistent protective components and changes in regulatory competence contribute to subsequent responsiveness.

The central question addressed in this review is how previous exposure could alter the functional configuration of the HSF/HSP network during recovery so that the response to a later challenge is changed. We use “recovery-associated state” to denote this experimentally accessible configuration of regulatory and proteostatic properties. Recovery is examined here in relation to the later challenge: the central issue is whether changes established between exposures modify HSF/HSP activation, protective capacity or response kinetics when stress recurs. The concept does not imply that a discrete molecular memory substrate exists or that HSF/HSP networks autonomously encode environmental history. Rather, it provides a framework for considering how transient and persistent changes in transcriptional competence, chaperone capacity, protein quality control, signalling, and related processes could interact over time.

This perspective connects two bodies of evidence that are often considered separately. The first comprises mechanistic studies of heat-induced transcriptional and proteostatic memory, where causal evidence is strongest. The second concerns priming and environmental history across different stresses, where altered subsequent responses are common but their mechanistic basis is more heterogeneous. Considering these areas together makes it possible to ask which principles established by thermomemory can reasonably be generalised and which remain specific to particular stresses, species, or molecular pathways.

The review first establishes the temporal and experimental distinctions needed to define HSF/HSP-dependent environmental memory. It then examines what the heat memory literature can currently support, assesses the extent to which these principles apply across broader environmental histories, and sets out experimental criteria for linking history-dependent responses to HSF/HSP processes while accounting for sustained protection and incomplete recovery. Finally, it develops a mechanistic framework in which HSF/HSP networks could generate a recovery-associated state through coordinated changes in transcriptional competence, chaperone capacity, client handling, and protein quality control.

## 2. Defining Environmental History and HSF/HSP-Dependent Environmental Memory

Plants can respond differently to the same stress depending on what occurred previously. However, terms such as acclimation, priming, and memory describe related but distinct phenomena. Acclimation generally refers to reversible adjustment to prevailing environmental conditions, whereas priming describes a prior exposure that modifies the response to a subsequent challenge; environmental memory is more specifically concerned with a history-dependent alteration in subsequent responsiveness that can be detected after the initiating stimulus has been removed [1,13]. The distinction is particularly important for HSF/HSP systems because many of their components are directly induced, modified, or engaged by stress and may remain altered during recovery. Thus, persistence of an HSF, HSP, or associated molecular feature should not be equated with memory unless it is linked to a measurable change in the response to a subsequent challenge.

The present review focuses on environmental memory within a generation, defined here as a change in the response of the same plant to a later environmental challenge following a previous exposure and an intervening recovery period. Memory transmitted across generations is outside the scope of this review.

### 2.1. The Recovery-Associated State

For the purposes of this review, environmental history refers to the sequence of environmental exposures experienced by a plant, including stress identity, intensity, duration, order, spacing, overlap, and the characteristics of the intervening recovery period. Environmental history is therefore not itself the memory state. Rather, it comprises the conditions that can generate a subsequent physiological or molecular state and influence how the plant responds to a later challenge.

The relevant focus is therefore the recovery-associated state rather than an individual HSF or HSP alone. Such a state can be understood as a configuration of regulatory and proteostatic properties established or remodelled during recovery. It may include changes in transcriptional competence, chromatin state, HSF abundance or activity, chaperone capacity, client occupancy, protein turnover, signalling, metabolism, and organellar homeostasis. These properties do not need to change with the same kinetics, and individual properties may return towards their prestress condition while others remain functionally relevant. This formulation does not imply that the recovery-associated state is a demonstrated discrete molecular entity. Operationally, the term describes measurable regulatory and proteostatic properties during recovery; the mechanistic hypothesis is that functional relationships among these properties contribute to subsequent responsiveness. Describing these properties does not, by itself, establish those relationships or their causal contribution to memory. Rather, it provides an operational framework for describing how persistent components and the lasting consequences of transient processes may influence a subsequent response.

This operational definition does not classify every state persisting after stress as memory. Unresolved physiological disturbance may influence the later phenotype, whereas a persistent protective component can support memory if its contribution to the subsequent response is demonstrated.

### 2.2. Memory Specific to a Stress, Priming, and Broader Environmental History

The clearest form of memory associated with HSF/HSP is memory specific to a stress, in which the initial and subsequent challenges are the same or closely related. Recurrent heat provides the principal experimental model because the stress can be precisely controlled and the recovery interval can be manipulated independently of the rechallenge. In this context, thermomemory encompasses both persistent molecular changes and altered physiological responsiveness, but these levels of evidence do not necessarily reflect the same underlying process.

Priming encompasses a broader range of history-dependent responses than the specific form of memory considered here. A plant may be primed by one stress and subsequently challenged by the same stress, a different stress, or a combination of stresses. Such responses are relevant to environmental history but should not automatically be equated with HSF/HSP-dependent memory. The persistence or expression of a primed state can involve several mechanisms, including shared signalling pathways, an altered metabolic state, enhanced antioxidant capacity, hormonal changes, and changes in protein homeostasis. Persistent damage may also influence the subsequent response and must be considered when interpreting the phenotype. HSF/HSP responses may contribute to these processes without necessarily constituting the mechanism by which environmental history is retained [1].

The distinction between a history-dependent phenotype and HSF/HSP-dependent environmental memory becomes particularly important when environmental history involves different stresses, such as drought followed by heat. Evidence that a first stress alters subsequent tolerance establishes a history-dependent phenotype, but does not by itself demonstrate that HSF/HSP components contribute causally to its persistence or expression. The strength of the mechanistic inference depends on linking a candidate HSF/HSP process to the altered response while accounting for changes in basal physiology and in the perception or severity of the subsequent stress.

### 2.3. Molecular Persistence, Functional Persistence, and Causal Memory

Evidence for environmental memory can arise at different biological levels, but these forms of evidence should not be treated as equivalent. Three levels are particularly important.

Molecular persistence refers to the continued presence or altered state of a molecular feature during recovery, such as the presence or sustained activity of an HSF or HSP, a post-translational modification, a chromatin state, a transcript, a metabolite, or a signalling activity. Molecular persistence establishes that a feature survives or remains altered after the initiating stress, but does not establish that it modifies the response to a later challenge.

Functional persistence refers to a measurable difference in the response to a subsequent challenge between plants with different prior environmental histories, when the plants are otherwise exposed to the same challenge conditions. It demonstrates that previous exposure has lasting functional consequences that become evident upon rechallenge. Functional persistence therefore provides stronger evidence for environmental memory than molecular persistence alone, but does not identify the molecular process responsible for the altered response.

Causal evidence requires showing that perturbing a candidate process alters the history-dependent response while accounting for effects on basal physiology and acute stress tolerance. A factor required for the initial stress response is not necessarily involved in memory. Conversely, a transient process may establish a lasting change even if the factor is no longer detectable at rechallenge. Persistence of a candidate factor at rechallenge is therefore neither necessary nor sufficient to demonstrate a causal role.

These forms of evidence are complementary rather than a strictly linear hierarchy: molecular persistence may accompany a functional phenotype without being causally required, while a transient process may contribute causally without leaving a persistent molecular marker.

Sustained protection and incomplete recovery require distinct interpretations within this framework. Sustained protection occurs when molecules, metabolites, physiological adjustments, or other consequences generated by the first exposure continue to provide direct protection during the second challenge. Such protection can contribute to environmental memory when its functional role after recovery is demonstrated; its persistence alone does not establish that role or imply additional changes in regulatory responsiveness. Incomplete recovery occurs when physiological or molecular disturbances caused by the first exposure remain unresolved at rechallenge and influence the later response.

### 2.4. Temporal Organisation of Memory

The processes contributing to a recovery-associated state can be considered across four partially overlapping phases: establishment, persistence or remodelling, functional expression, and resetting. Establishment encompasses processes initiated by the first exposure that create conditions for altered future responsiveness. Persistence or remodelling refers to regulatory and proteostatic changes that are maintained or reorganised during recovery. Functional expression occurs when the subsequent challenge reveals the altered response, whereas resetting encompasses processes that progressively return the system towards its prestress configuration.

These phases should not be interpreted as rigid sequential stages or as periods controlled by distinct sets of molecules. The same factor may contribute to more than one phase, and the temporal role of a process must be established experimentally rather than inferred solely from its abundance.

Recovery should therefore be considered part of the environmental sequence rather than simply an interval between two treatments. Varying its duration can help distinguish persistence, decay, remodelling, and resetting of the response and can reveal how the consequences of the initial exposure influence subsequent responsiveness.

**Figure 1:**
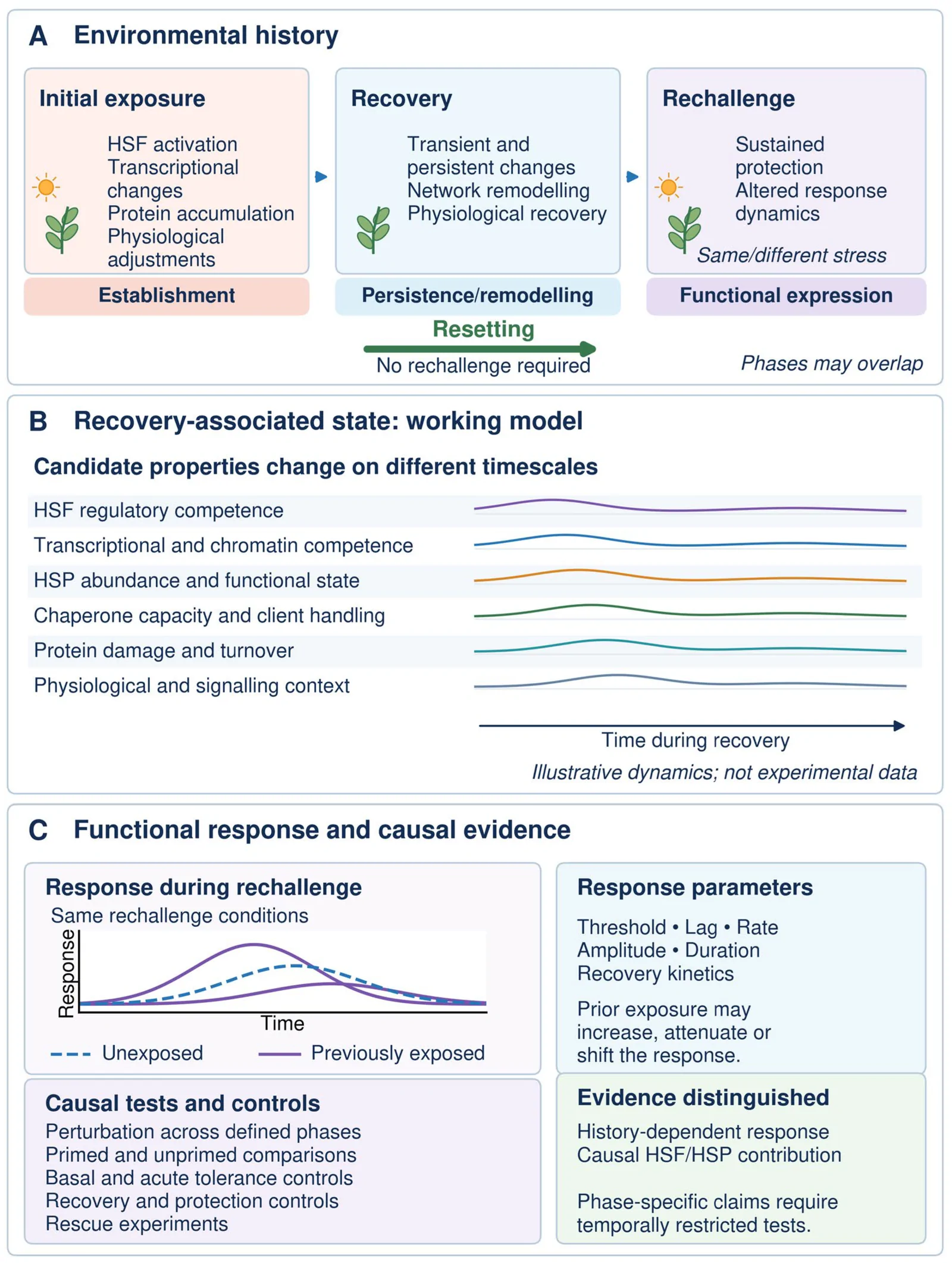
Temporal and experimental framework for HSF/HSP dependent environmental memory. (**A**) Initial exposure, recovery and rechallenge, with potentially overlapping functional phases. Resetting may occur during recovery without rechallenge; the arrow does not define a fixed temporal window. (**B**) The recovery-associated state is a heuristic working model, not a demonstrated discrete cellular entity. Candidate properties may change on different timescales. (**C**) Previous exposure may increase, attenuate or shift the response to the same rechallenge. Purple curves represent alternative responses of previously exposed plants; the dashed blue curve represents previously unexposed plants. A greater response amplitude does not necessarily indicate improved tolerance. Causal inference requires appropriate perturbations and controls; phase specific claims require temporally restricted tests. All curves are schematic. HSF, heat shock factor; HSP, heat shock protein.

Figure 1 summarises the environmental sequence, candidate recovery-associated properties, and experimental distinctions used throughout this review.

## 3. From Heat Memory to Environmental History Integration: What the HSF/HSP Evidence Establishes

Heat stress currently provides the strongest experimental framework for determining whether processes associated with HSF/HSP contribute causally to persistent changes in subsequent stress responsiveness. Its value lies not simply in the number of studies available, but in the diversity of molecular levels at which causal contributions have been demonstrated. These include HSFs, chaperones, protein turnover, regulation involving chromatin, and transcriptional machinery. The evidence therefore provides a basis for examining how these processes support persistent responsiveness and whether their contributions can be understood within an integrated recovery-associated state.

### 3.1. Experimental Evidence for HSF/HSP Contributions to Heat Memory

Early genetic studies provided some of the first evidence that acquired thermotolerance can persist beyond the initiating heat exposure, raising the question of which processes during recovery sustain this history-dependent response. The requirement for HSA32 illustrates this temporal dependence: loss of HSA32 compromises maintenance of acquired thermotolerance after prolonged recovery but has little effect when thermotolerance is assessed after a shorter recovery interval [3]. HSFA2 likewise contributes to the extension of acquired thermotolerance [14], indicating that the molecular requirements for prolonged heat tolerance differ from those governing the immediate heat response. At the level of protein homeostasis, the functional interaction between HSA32 and HSP101 was shown to prolong heat acclimation memory at the post-transcriptional level, providing an early link between HSP-dependent proteostasis and persistent heat responsiveness [15].

Subsequent studies linked heat memory to sustained transcription and chromatin modifications. HSFA2 associates transiently with memory loci during early recovery, whereas sustained H3K4me3 enrichment and transcriptional memory depend on HSFA2 and outlast its association with these loci [16]. HSFA2 and HSFA3 form heteromeric complexes that promote transcriptional memory, with HSFA3 required for sustained expression of memory-associated genes after return to normal temperatures. Genetic analyses also implicate HSFA2 and HSFA3 in sustained enrichment of histone H3 lysine 4 trimethylation (H3K4me3), a chromatin mark associated with transcriptionally active or permissive chromatin, at memory loci during recovery. Their contributions to sustained expression and enhanced reinduction are not identical. Whereas both factors support sustained expression during recovery, enhanced reinduction of the memory genes examined after a second heat treatment depends on HSFA2 but remains intact in hsfa3 mutants [5]. A complementary requirement for sustained memory-associated gene expression is provided by BRU1, whose loss affects transcriptional persistence while leaving the acquisition of thermotolerance largely intact [17]. FORGETTER1 (FGT1) provides complementary evidence for nucleosome remodelling. FGT1 interacts with the chromatin remodelers BRAHMA, CHR11 and CHR17, and its loss accelerates the restoration of nucleosome occupancy at tested memory loci. Mutant plants show premature loss of sustained gene expression and reduced thermotolerance after prolonged recovery, while basal thermotolerance and the initial acquisition of thermotolerance remain largely unaffected [18]. Genome-wide analyses further identified distinct genomic and epigenomic features associated with heat memory genes and characterised HSFA2 and HSFA3 binding patterns at genes exhibiting transcriptional memory, including an association between sustained transcription and H3K4me3 enrichment during recovery [19].

Locus-targeted epigenome editing provides a more direct test of chromatin involvement. Heat-inducible recruitment of dCas9–JMJ18 to APX2 reduced H3K4me3 enrichment and impaired the enhanced transcriptional reinduction normally observed after recurrent heat. However, the catalytically inactive fusion also affected chromatin methylation and, to a lesser extent, transcriptional memory, so the phenotype cannot be attributed exclusively to demethylase activity [20]. In a complementary study, mutations in the H3K4 methyltransferases SDG25 and ATX1 reduced H3K4me3 enrichment and the sustained expression of a subset of heat-responsive genes during recovery, but did not produce a detectable defect in the maintenance of acquired thermotolerance under the conditions tested [21]. This illustrates why molecular effects during recovery and physiological memory must be assessed separately.

Chromatin regulation also involves removal of repressive marks. JUMONJI demethylases support sustained reduction in histone H3 lysine 27 trimethylation (H3K27me3) at HSP22 and HSP17.6C, facilitating their activation upon recurrent heat after three days. Genetic and inducible-expression analyses support a functional contribution of this pathway to heat acclimation memory [22].

The Mediator complex, a conserved transcriptional coactivator that facilitates communication between transcription factors and the basal transcriptional machinery, adds another layer to this regulatory architecture through its kinase module. This module includes CDK8 and MED12. CDK8 contributes to sustained expression during recovery and enhanced reinduction upon recurrent heat, whereas the MED12 allele examined primarily impairs enhanced reinduction. Interaction assays and reduced CDK8 occupancy at APX2 and MIPS2 in hsfa2 plants support recruitment through HSFA2. CDK8 kinase activity is required for transcriptional memory, and polymerase occupancy and nascent transcript measurements suggest that it promotes productive RNA polymerase II transcription; its precise contribution to elongation, termination or recycling remains unresolved [7]. Together, these findings indicate that sustained transcriptional responsiveness depends on regulatory mechanisms that extend beyond the initial activation of heat-responsive genes.

Protein homeostasis provides a complementary level of temporal regulation. Heat priming induces both HSP21 and FtsH6, but their effects during recovery differ: persistent HSP21 supports thermomemory, whereas FtsH6 promotes its subsequent decline. HSP21 abundance is initially similar in wild-type and ftsh6 seedlings but remains higher in the mutant from 24 h into recovery onwards [4]. HSFA2 binds the FtsH6 promoter during recovery and activates its expression, thereby supporting a pathway that limits HSP21 persistence. Consistently, HSFA2 overexpression produces greater HSP21 accumulation and stronger thermomemory in the ftsh6 background than in wild type [6]. HSFA2 can therefore contribute to both the maintenance and resetting of thermomemory through different downstream targets, illustrating how the same regulatory factor can support distinct functions during recovery.

The HSP90.1-associated co-chaperone ROF1 also contributes to prolonged thermotolerance: rof1 mutants show reduced expression of HSFA2-regulated small HSP genes during recovery and impaired survival when severe heat follows acclimation after 24–48 h [23]. Selective autophagy provides another pathway controlling memory duration: NBR1 mediates turnover of HSP90.1 and ROF1 during recovery, attenuating gene expression regulated by HSFA2 and limiting heat memory [24]. Subsequent work identified NBR1 domains involved in recognition of non-ubiquitinated ROF1, providing mechanistic detail on cargo recognition in this pathway [25].

Recent work has extended this temporal view from HSF abundance and transcriptional activity to the molecular properties of HSF proteins themselves. Promoter and domain swap experiments showed that placing HSFA1D under the HSFA2 expression pattern is insufficient to restore physiological heat memory, while domain swap analysis identified protein regions, including the oligomerisation domain, that contribute to the function of HSFA2 specific to memory [8]. These findings indicate that HSF abundance or expression pattern alone does not capture all determinants of memory competence. At another level of HSF organisation, two distinct prion-like domains, PrD1 and PrD2, in sensory HSFs contribute to heat activation and subsequent responsiveness. In HSFA1a, PrD1 supports temperature-sensitive interaction with HSP70, whereas PrD2 supports heat-induced formation of transcriptionally active foci. Evidence for memory comes from a separate comparison: after a 5 min priming treatment at 37 °C and three days of recovery at 22 °C, a second heat treatment elicited enhanced HSP70 and APX2 expression, which was lost upon PrD2 deletion. Chromosome conformation capture also detected stronger promoter–enhancer contacts upon rechallenge in primed plants; this enhancement was absent in the PrD2 deletion line. These findings distinguish acute HSF activation from enhanced reinduction after recovery, but do not establish continuous persistence of foci or elevated promoter–enhancer contacts throughout recovery [9]. These observations implicate HSF protein state and higher-order organisation in persistent heat responsiveness, but they do not establish HSF assemblies as an autonomous or universal memory substrate.

Protein quality control can also preserve HSF functionality during heat stress. In vitro biochemical assays support a chaperone function of Universal Stress Protein 1 (USP1) in stabilising HSFA2 against heat-induced denaturation. Loss of USP1 impairs both acquired thermotolerance and heat stress memory. Plants lacking USP1 show reduced expression of genes associated with heat memory and lower enrichment of H3K4me3 at memory loci. These observations are consistent with a contribution of HSFA2 protection to memory, but do not establish that reduced H3K4me3 enrichment results specifically from impaired HSFA2 stabilisation. Neither direct chromatin regulation by USP1 nor its interaction with HSFA2 in vivo was demonstrated [26].

HSF contributions to thermomemory can also involve tissue-specific hormonal regulation. In the *Arabidopsis* shoot apical meristem, HSFA7b regulates ethylene homeostasis through targets involved in ethylene signalling and biosynthesis, including ETHYLENE-INSENSITIVE 3 and ethylene biosynthesis repressors. Loss of functional HSFA7b compromises meristem activity following thermopriming, linking HSF-dependent regulation to the maintenance of growth in this tissue [27].

Table 1 summarises the principal experimental evidence, distinguishing the function implicated by each study, the supporting observations, and the limits of mechanistic inference. The functions listed reflect the findings and interpretation of the respective studies; they do not necessarily indicate causal involvement demonstrated by perturbation restricted to a particular phase.

### 3.2. Temporal Organisation, Resetting, and Limits of the Heat Memory Evidence

Heat memory studies identify contributions to both persistence and attenuation during recovery. Transcriptional regulation, chaperone functionality, and protein turnover operate at different biological levels, making their temporal coordination an important unresolved question. At the chromatin level, reduced histone H3.3 turnover at heat memory genes provides a mechanism supporting the retention of H3K4me3 during recovery, illustrating that persistence can depend on the dynamics of component replacement [29].

Regulated protein turnover demonstrates that resetting can actively limit memory duration. However, declining protein abundance alone cannot distinguish passive decay from regulated removal or establish that either process controls memory duration. Evidence for active resetting requires perturbing the proposed regulatory process and testing whether this changes the persistence of the history-dependent phenotype, while accounting for effects on physiological recovery and acute stress tolerance. Genetic evidence implicating FtsH6 and NBR1-mediated selective autophagy supports regulated attenuation of thermomemory in these specific pathways [4,6,24]. Autophagy also complements FtsH6 in controlling HSP21 turnover during recovery. Combined disruption of autophagy and metalloprotease activity increases HSP21 accumulation and prolongs thermomemory, indicating that distinct degradation pathways can converge on the persistence of the same protective component [30]. These findings do not imply that all declining HSF/HSP components undergo active resetting.

Attenuation can also involve transcriptional feedback. SMXL4 and SMXL5 repress HSFA2 transcription, whereas HSFA2 binds their promoters and activates their expression during recovery, forming a negative feedback circuit [31]. This identifies regulated control of HSF output after heat, although a recovery-specific requirement for this circuit in resetting thermomemory remains to be established. Evidence concerning the return of transcription and chromatin marks towards baseline comes from CSN5A perturbation. In *Arabidopsis*, a hypomorphic mutation in the COP9 signalosome subunit CSN5A delayed the return of APX2 and HSP22 transcript levels to baseline after recurrent heat and altered H3K4me3 dynamics at these loci [32]. These findings implicate CSN5A in post-stress resetting, although they do not establish a direct mechanism linking CSN5A to HSF activity.

The heat memory literature nevertheless has important limits. The available evidence spans distinct levels of organisation, from transcriptional regulation and changes in chromatin to chaperone persistence, HSF functionality, and physiological thermotolerance, and these levels should not be assumed to represent a single molecular state. Moreover, constitutive perturbations and measurements across recovery intervals do not necessarily resolve the phase during which a factor is causally required. The strongest conclusion supported by the available evidence is therefore that selected processes associated with HSF/HSP contribute to distinct molecular and functional aspects of heat memory. How these processes interact within an integrated recovery-associated state remains unresolved.

### 3.3. From Recurrent Heat to Broader Environmental Histories

Environmental histories extend beyond recurrent heat to different stresses, repeated episodes separated by recovery, and sequences in which multiple stresses overlap. Examining these histories tests the applicability of principles identified in thermomemory while recognising that the underlying mechanisms may differ.

Broader histories may engage sustained protection, persistent signalling, metabolic adjustment, and other forms of acclimation, while unresolved physiological disturbance may also influence later performance [1]. Their mechanistic interpretation therefore depends on the stress sequence and the recovery conditions.

An important first question is whether the HSF-dependent mechanisms identified in *Arabidopsis* heat memory extend to other plant species. Evidence from barley indicates that core components of this response can be conserved while their downstream implementation diverges. HvHSFA2 and HvHSFA3 are required for heat memory, but their loss broadly attenuates transcriptional responses to recurrent heat rather than selectively disrupting the *Arabidopsis*-like memory programme. For example, the barley memory genes examined did not show increased H3K4me3 enrichment at 4, 24 or 48 h after priming, contrasting with the sustained enrichment observed at *Arabidopsis* memory loci [28]. Thus, conservation of an HSF-dependent memory function does not necessarily imply conservation of an identical molecular circuit.

Conservation also extends to proteostatic components. In rice, posttranscriptional feedback between HSP101 and HSA32 prolongs acquired thermotolerance, supporting conservation of this regulatory relationship beyond *Arabidopsis*. Moreover, the rice cultivar with greater basal thermotolerance showed weaker long-term acquired thermotolerance, demonstrating that these traits need not vary together [33].

Recent evidence in tomato further extends causal HSF involvement beyond *Arabidopsis*. Following a five-day recovery interval, SlHSFA2 CRISPR mutants showed impaired thermal priming and altered enhanced reinduction of memory genes. SlHSFA2 also interacts with ethylene response factors that enhance activation of selected targets, including SlBAG6, identifying a regulatory module associated with transcriptional thermal memory in this crop [34].

Functional evidence is also available in tall fescue (*Festuca arundinacea*). CRISPR-mediated disruption and overexpression of FaHSP17.8-CII altered the protection of photosystem II and the control of reactive oxygen species following heat acclimation and subsequent heat stress [35]. These experiments support a contribution of this small HSP to priming-associated protection, although they do not isolate its requirement during recovery. Proteomic analyses provide complementary evidence in tall fescue. Following repeated heat priming, HSP21 and other chloroplast-associated proteins showed increased abundance both before and during the subsequent heat challenge, alongside improved photosystem II performance. These observations identify candidate protective components, although their causal contribution was not tested by functional perturbation [36].

A greater mechanistic uncertainty emerges when the initial and subsequent stresses differ. Shared HSF/HSP involvement is biologically plausible: in *Arabidopsis*, HSFB1 directly activates HSP101, which contributes genetically to drought tolerance [37]. This establishes a function outside heat stress, but does not demonstrate memory or cross stress priming.

In durum wheat, dehydration pretreatment enhances subsequent thermotolerance and is accompanied by changes in the expression of several HSP genes, but no separate recovery interval is included between dehydration and heat [38]. This demonstrates acquisition of cross stress thermotolerance without establishing persistence after recovery. Earlier studies in spring and winter wheat showed that drought priming during vegetative growth can improve physiological performance and nitrogen use efficiency under subsequent drought and heat stress during grain filling [39,40]. In a subsequent study, drought priming at the jointing stage improved tolerance to heat imposed after anthesis in two wheat cultivars. Reduced membrane damage was accompanied by changes in antioxidant activity, TaHSF expression and metabolite profiles, extending the evidence for cross stress priming to coordinated physiological and molecular responses [41]. Comparable cross stress priming has been reported in rice, where water deficit during vegetative growth improved performance under heat at anthesis and was accompanied by increased expression of OsHSP16.9A, OsHSP70.1 and OsHSP70.6 [42]. In chickpea, three days of drought pretreatment followed by six days of recovery altered the expression of HSF and HSP genes and associated miRNAs measured after a subsequent heat treatment [43].

Cross stress effects can also depend on the response measured. In tomato, thermopriming followed by salt treatments altered leaf phenolic and flavonol accumulation but did not prevent the salt-induced delay in vegetative growth [44]. These results extend the range of documented responses to sequential stresses without establishing an HSF/HSP-dependent memory mechanism.

More recently, repeated drought priming in creeping bentgrass was shown to improve tolerance to subsequent drought or heat after recovery. Two drought cycles were each followed by one day of rewatering. Gamma-aminobutyric acid (GABA) accumulation was already elevated after the second recovery, whereas enhanced NO accumulation was reported after seven days of subsequent drought or heat. HSF, HSP and dehydrin transcripts were measured after the second recovery and at 3 h and 7 d of the subsequent stress, revealing responses that depended on the gene, stress and sampling time. These observations distinguish changes present before rechallenge from altered responses during the later stress, without establishing a causal HSF/HSP mechanism of persistence [45]. Together, these studies show that previous water deficit can modify responses to subsequent drought or heat, potentially through interacting proteostatic, metabolic and signalling processes.

In soybean, sequential water deficit and waterlogging alter subsequent physiological responses and recovery capacity, with outcomes depending on stress identity and order [46]. This extends the environmental history framework to contrasting forms of water stress, although HSF/HSP involvement was not tested.

Combined stress experiments provide a further distinction between stress integration and environmental memory. In *Arabidopsis*, HSFA4A contributes to tolerance to simultaneous heat and salt stress through phosphorylation by mitogen-activated protein (MAP) kinases and regulation of stress-responsive genes, including HSP17.6A, WRKY30, and ZAT12 [47]. This study provides mechanistic evidence for HSF participation in combined stress signalling, but does not establish whether the associated regulatory changes persist after recovery or influence a subsequent challenge.

A related question concerns the effects of recurrent combined stress. In wheat, moderate combined heat and deficit irrigation imposed at stem elongation or booting was followed by a more intense combined challenge after anthesis. Previous exposure modified subsequent responses, with priming effects depending on genotype and developmental stage and being particularly evident in yield traits [48]. These results extend the evidence for priming to recurrent combined stresses, although the mechanisms underlying persistence and any contribution of HSF/HSP networks remain unresolved.

The transition from thermomemory to broader environmental histories is summarised in Table 2. The table compares the type and strength of evidence available across environmental sequences and includes simultaneous heat and salt as a comparator for stress integration. The central question is whether previous exposure produces regulatory or proteostatic changes that contribute to the later response, and how those contributions depend on recovery.

Across species, conservation of HSF/HSP sequences or stress-induced expression should therefore be distinguished from conservation of a causal memory mechanism. Most mechanistic evidence considered here derives from *Arabidopsis*; genetic studies in barley, rice and tomato nevertheless demonstrate contributions of HSFs or HSPs to prolonged heat responsiveness, while revealing both conserved regulatory relationships and species-specific features [28,33,34]. In contrast, the cross stress and recurrent combined stress studies considered here primarily establish physiological, agronomic or transcriptional responses, without demonstrating causal HSF/HSP involvement in memory. Moreover, genetic perturbations restricted to recovery or rechallenge remain scarce, limiting the assignment of phase-specific functions even where genetic involvement has been established.

## 4. Experimental Criteria for Establishing HSF/HSP-Dependent Environmental Memory

Establishing HSF/HSP involvement in environmental memory requires a defined environmental history, a measurable alteration in the response to a subsequent challenge, adequate characterisation of the intervening recovery period, and evidence linking the history-dependent phenotype to a specific regulatory or proteostatic process. These requirements apply both to recurrent exposure to the same stress and to more heterogeneous histories involving sequential or combined stresses. For HSF/HSP systems, the central experimental task is to determine whether a candidate process contributes to the effect of previous exposure, rather than merely participating in the acute response to the subsequent challenge.

The evidential framework used throughout this review is summarised in Table 3. Rather than defining memory through the presence of a particular molecular marker, the framework distinguishes the evidence required to identify a history-dependent phenotype and establish the causal contribution of HSF/HSP processes.

### 4.1. Defining Environmental History and Characterising Recovery

To establish whether a previous stress exposure modifies the response to a subsequent challenge, the environmental history must first be clearly defined. Experiments should specify the identity, intensity, duration, sequence, and spacing of stress exposures, together with the duration and conditions of the intervening recovery period. These variables determine which consequences of the initial treatment may remain relevant when the subsequent challenge is imposed.

Recovery duration should be varied whenever feasible rather than defined by a single priming and rechallenge interval. Comparing different intervals can reveal whether an effect decays, persists, or changes over time. This is particularly relevant to HSF/HSP systems, whose components can differ in their persistence and turnover kinetics. In *Arabidopsis*, transcript and protein responses during recovery depend on the duration of the initial heat treatment, illustrating that the molecular response evolves according to both the initial exposure and the time elapsed since it [49].

The recovery period should be characterised independently of the subsequent challenge, using measurements that distinguish ongoing acute responses from changes persisting after stress withdrawal. Sampling should capture both early and later recovery, because a transient process may have consequences that remain relevant after the initiating component has declined.

Recurrent exposure to the same stress should also be distinguished from sequential exposure to different stresses. Recurrent heat provides a tractable framework in which the priming stimulus, recovery interval, and subsequent challenge can be varied independently. Histories involving different stresses, such as drought followed by heat, require additional consideration because changes in water status, metabolism, redox balance, hormonal signalling, or cellular damage may influence the subsequent response independently of HSF/HSP processes. The same experimental principles apply to both memory specific to a stress and broader environmental histories, although experiments involving different stresses may require a wider assessment of the physiological state during recovery.

### 4.2. Demonstrating History-Dependent Functional Change

Defining the environmental history and characterising recovery are not sufficient to establish environmental memory. Experiments must also demonstrate that previous exposure modifies the response to a subsequent challenge. The critical comparison is between plants with different exposure histories subjected to the same triggering conditions. Where feasible, a factorial design should include plants receiving neither treatment, the priming treatment alone, the challenge alone, or both treatments. These groups help distinguish the effects of priming on basal physiology from its effects on the response to the subsequent challenge.

History dependence is demonstrated most directly when plants with different exposure histories respond differently during or after rechallenge. Physiological outputs may include survival, growth, photosynthetic performance, membrane integrity, or damage accumulation, depending on the stress and tissue examined. Molecular outputs may include transcriptional responses, HSF activation, HSP accumulation, protein turnover, or other stress-responsive processes. A modified response may therefore be physiological, molecular, or both, but its interpretation depends on the relationship between the initial exposure, the recovery period, and the subsequent challenge.

Dynamic measurements can reveal history-dependent differences that a single endpoint would miss. Depending on the experimental system, relevant variables include the threshold for HSF activation, response lag, rate of transcription or protein accumulation, response amplitude and duration, and the kinetics of damage clearance. These variables are pertinent to HSF/HSP networks because their functional output depends not only on component abundance but also on the timing of HSF activation, chaperone availability, client load, and protein turnover. Plants that reach similar final HSP abundance may nevertheless differ in how rapidly, or under what conditions, they activate the response. Such differences can reveal altered response capacity even when endpoint measurements appear similar [50].

In tomato, heat priming was associated with improved photosynthetic performance and water relations during subsequent heat, alongside distinct transcriptional responses detected at 6 and 12 h after challenge onset, illustrating the value of combining physiological and molecular measurements at multiple time points [51].

Response measurements should be interpreted alongside each group’s physiological state immediately before rechallenge, because pre-existing differences may influence subsequent performance.

Altered HSF activation or HSP accumulation during rechallenge may reflect an acute response rather than a mechanism mediating the effect of previous exposure. Establishing that contribution requires functional perturbation and appropriate controls for basal physiology and acute stress tolerance, as summarised in Table 3.

### 4.3. Assessing Sustained Protection and Incomplete Recovery

A history-dependent phenotype may involve sustained protection, incomplete recovery, or a change in the capacity to respond to a subsequent challenge. These processes are not mutually exclusive, but they require different experimental interpretations. Sustained protection occurs when components induced by the initial stress remain directly protective during rechallenge. Persistent HSP abundance, for example, can contribute to thermomemory when its role in maintaining later thermotolerance is demonstrated. However, abundance alone does not establish this functional contribution or show that other regulatory properties have changed.

Incomplete recovery presents a distinct problem. If cellular damage, metabolic imbalance, altered growth, changes in water status, or disrupted homeostasis persist when the second stress is imposed, the resulting phenotype may reflect the continuing consequences of the first exposure. Recovery can also differ among physiological processes, so apparently normal performance in one measure does not establish that the plant has returned to its previous state in all relevant respects. This issue is particularly important in experiments involving different stresses, where the initial treatment may alter several physiological systems and thereby influence the severity or perception of the subsequent stress.

Distinguishing these contributions requires combining physiological measurements immediately before rechallenge with response kinetics during and after it, rather than relying solely on final phenotypes. Relevant measurements may include growth, photosynthetic or respiratory performance [52], cellular damage, water status, and selected metabolic or hormonal parameters. Their selection should reflect the initial stress, the tissue and developmental stage examined, and the expected consequences of the treatment. Responses should be evaluated relative to each group’s prechallenge baseline, while assessing whether differences in water status, tissue temperature or other relevant variables alter the effective severity of the subsequent stress. Similar final performance can conceal different response kinetics, whereas a faster response can arise from unresolved physiological differences.

No universal physiological threshold defines complete recovery, and detectable differences from untreated plants do not automatically exclude memory. Rather, the objective is to identify which consequences of the initial exposure persist and whether they account for the phenotype observed after rechallenge. Neither apparently restored baseline physiology nor altered response kinetics alone establishes memory; their interpretation requires appropriate recovery controls and, for causal HSF/HSP attribution, functional perturbation.

Recovery duration should therefore be treated as an experimental variable rather than as a fixed criterion for memory. A phenotype that diminishes as recovery proceeds may reflect the loss of a protective component, the resolution of physiological disturbance, or active resetting of a memory-associated process. Persistence after selected stress-induced changes have subsided provides a basis for investigating other mechanisms, but does not demonstrate that all direct effects of the initial stress have resolved. The importance of recovery timing is illustrated by recurrent heat experiments in winter oilseed rape, in which the interval between a mild and a subsequent intense heat event influenced seed biomass and quality [53]. This study highlights the influence of recovery timing on later stress outcomes, but does not establish an HSF/HSP-dependent memory mechanism.

Sustained protective capacity and altered regulatory responsiveness may both contribute to the phenotype. Their relative importance should be assessed alongside unresolved physiological disturbance rather than treating sustained protection as incompatible with memory.

### 4.4. Establishing Temporal and Causal HSF/HSP Involvement

Establishing a causal contribution to HSF/HSP-dependent environmental memory requires perturbing a candidate process at the phase in which it is hypothesised to act. Perturbation confined to the initial stress may reveal a role in acute resistance without establishing a contribution to memory. Manipulation during recovery can test whether a process is required to establish or maintain altered responsiveness, whereas perturbation during rechallenge can determine whether it is necessary for expression of the history-dependent phenotype.

Temporally restricted experiments provide greater resolution than constitutive perturbations, which may affect several phases of the response. Comparing perturbations across recovery intervals can further reveal whether the requirement for a factor changes as recovery proceeds. This approach helps distinguish contributions to establishment, persistence or remodelling, functional expression, and resetting without assigning temporal functions solely from measurements of abundance.

Causal inference is strengthened when perturbation changes the history-dependent phenotype while effects on basal physiology and acute stress tolerance are independently assessed. Where feasible, the effect of a perturbation should be compared between primed and unprimed plants exposed to the same challenge, rather than inferred solely from the performance of primed plants. Loss of function, gain of function, manipulation of specific domains, inducible perturbation, and temporally restricted interference offer different levels of resolution. Complementary rescue experiments or independent perturbation strategies can strengthen the inference and help exclude indirect effects.

Heat-inducible manipulation of miR156 altered heat memory without the developmental changes associated with constitutive manipulation, helping to exclude pre-existing shoot architecture as the cause of the phenotype [54]. Complementary evidence comes from CDK8, which promotes transcriptional memory while being largely dispensable for initial heat-induced gene activation, separating its contribution to memory from primary transcriptional induction [7].

Necessity and sufficiency should nevertheless be distinguished: showing that a factor is required for a phenotype does not establish that it is sufficient to generate that phenotype, nor that it constitutes the memory mechanism on its own.

Resetting can be investigated by testing whether the manipulation of a recovery process changes the duration or disappearance of the history-dependent phenotype. Measurements across several recovery intervals should assess whether the perturbation prolongs or shortens the effect of previous exposure while accounting for changes in physiological recovery and acute stress tolerance. Such experiments can distinguish active regulation of memory duration from the observed decline in a molecular marker.

A further objective is to determine whether coordinated manipulation of interacting regulatory and proteostatic variables can reproduce defined features of a history-dependent phenotype. This is a proposed strategy for testing the model. A successful experiment would support the contribution of the manipulated relationships under the conditions tested, but would not establish reconstruction of the complete cellular state or exclude alternative mechanisms producing the same phenotype. Such reconstruction is not required to demonstrate the causal contribution of an individual component. Table 3 summarises the distinct inferences supported by these experimental approaches.

## 5. How HSF/HSP Networks Could Generate a Recovery-Associated State

Genetic and mechanistic studies establish that selected processes associated with HSF/HSP can contribute causally to heat memory and that environmental history can alter subsequent stress responsiveness. These studies do not, however, provide an experimentally complete reconstruction of how these processes interact to generate a persistent recovery-associated state. They establish contributions of selected components, but do not resolve the combined temporal relationships among regulatory and proteostatic processes or demonstrate that experimentally recreating those relationships reproduces the later phenotype. The framework developed here should therefore be regarded as a mechanistic synthesis and heuristic working model, not as a demonstrated discrete cellular entity or an experimentally established universal mechanism.

The central proposition is that environmental history could be retained through changes in the configuration and coupling of regulatory and proteostatic processes during recovery. The mechanistic question is how these relationships influence subsequent HSF activity, protein homeostasis, and stress responsiveness.

### 5.1. HSF Regulatory Competence and Proteostatic Capacity

HSF activity is determined by more than transcript or protein abundance. Relevant variables include protein stability and interactions with chaperones [55], as well as post-translational modification, subcellular localisation, and DNA binding [56]. HSF competence also depends on interactions with coregulators [7] and higher-order organisation [9]. For example, phosphorylation of HSFA1d by BRASSINOSTEROID INSENSITIVE 2 (BIN2) restricts its nuclear localisation and DNA-binding activity, demonstrating direct regulation of HSF functional competence [56]. In wheat, the modification of TaHsfA1 by small ubiquitin-related modifier (SUMO) promotes its transcriptional activation capacity, whereas prolonged heat reduces SUMOylation and attenuates downstream responses [57]. These examples establish regulation of HSF activity by post-translational modifications, but do not determine whether previous exposure changes HSF modification states during a later rechallenge. Establishing this contribution requires comparing modification dynamics in primed and unprimed plants and testing their effects on subsequent responsiveness. Heat memory studies show that HSF complex formation and protein architecture influence memory competence [5,8]. For the framework developed here, these findings imply that similar HSF abundance need not correspond to equivalent capacity to sustain or reactivate a transcriptional programme.

Hormonal signalling can also sustain the transcriptional programme. During heat memory in *Arabidopsis*, brassinosteroids promote accumulation of dephosphorylated BRI1-EMS-SUPPRESSOR 1 (BES1), which directly activates HSFA3 and APX2 and supports sustained expression of memory-associated genes [58].

The higher-order organisation and maintenance of protein integrity provide complementary routes through which HSF competence could be regulated. PrD2-dependent enhancement of transcriptional reinduction after recovery links sensory HSF organisation to a later response [9]. Separately, the in vitro stabilisation of HSFA2 by USP1, together with the impaired heat memory of usp1 mutants, supports a possible contribution of HSF protein protection [26]. These findings identify protein organisation and integrity as candidate determinants of regulatory competence, without establishing that either remains altered throughout recovery.

RNA processing also responds to prior heat exposure. Following four days of recovery from heat priming, *Arabidopsis* plants showed more efficient splicing after a subsequent severe heat treatment than non-primed plants, linking RNA processing to history-dependent responsiveness [59]. In *Arabidopsis*, the splicing factor RNA-DIRECTED DNA METHYLATION 16 (RDM16) supports efficient splicing of multiple HSF transcripts, while cooperative condensation of its protein isoforms enhances acquired thermotolerance [60]. The RDM16 thermotolerance assays used a recovery interval of only 30 min and therefore do not establish whether this factor contributes to memory maintained over several days.

Chemical RNA modifications provide an epitranscriptomic layer distinct from alternative splicing. In *Arabidopsis*, disruption of the N^6^-methyladenosine (m^6^A) demethylase AtALKBH10B increased m^6^A enrichment in floral transcripts, impaired activation of heat-responsive genes and reduced fertility under heat stress [61]. These findings link RNA methylation to heat responsiveness, but the study did not use a priming–recovery–rechallenge design. Whether RNA modifications alter HSF/HSP transcript stability, translation or reinduction after previous exposure therefore remains a question to test within this framework.

Recent work provides a further mechanistic example of the coupling between proteostasis and HSF activity. The loss of AtDJB3, a class II J-domain protein induced by heat, impairs HSC70-1 recruitment to protein aggregates and leaves HSFA1d associated with HSC70-1 in the cytoplasm. These observations support a model in which AtDJB3 promotes HSF activation by redirecting HSC70-1 towards aggregates. This process supports HSFA1d association with promoters of heat-inducible genes and contributes to thermotolerance [62]. Although thermomemory was not examined, this mechanism suggests how changes in proteostatic demand could influence subsequent HSF responsiveness.

The same principle applies to the HSP system. Total HSP abundance provides only a partial estimate of proteostatic capacity. Work on plastid HSP90C illustrates how structural features of a chaperone regulate its activity and client binding [63]. J-domain proteins also contribute to aggregate remodelling [64]. These functions provide plausible routes through which proteostatic capacity could be remodelled during recovery. Whether such changes contribute causally to environmental memory remains to be established.

The relevant quantity for a recovery-associated state may therefore be the relationship between regulatory competence, available chaperone capacity, and the proteostatic demands imposed by the cellular environment. Persistent HSP accumulation could contribute to this relationship, but abundance alone would not describe how much capacity remains available or how effectively it can be deployed during a subsequent challenge.

### 5.2. Client Handling, Protein Quality Control, and Recovery

The functional consequences of an altered HSF state depend on the capacity of the proteostasis network to process proteins generated or damaged during and after stress. Chaperone systems, disaggregation machinery, autophagy [65], and proteolytic pathways therefore provide potential links between protein damage during stress and the capacity to respond to a subsequent challenge. HSP101 contributes to the solubilisation and processing of aggregated proteins, whereas the J-domain protein AtDJB3 participates in the remodelling of protein aggregates, illustrating that recovery depends on coordinated control of damaged and misfolded protein populations rather than on chaperone abundance alone [64,66]. Evidence from defined clients illustrates this cooperation: reducing class I or class II small HSP expression impairs the return of heat-sensitive translation elongation factor eEF1B subunits to the soluble fraction during recovery, a process that also depends on HSP101 [67]. Proteomic comparisons of rof1, rof2 and double mutants further show that perturbing HSP90-associated co-chaperones alters the accumulation of chaperones and protein quality control components during heat acclimation [68]. Genetic evidence also implicates HSP70–HSP90 organising proteins (HOPs): the *Arabidopsis hop1 hop2 hop3* mutant showed impaired acquired thermotolerance after two days of recovery and, in separate heat treatments, increased accumulation of insoluble and ubiquitinated proteins [69]. These results connect co-chaperone function with both proteostasis and sustained thermotolerance, without resolving whether HOP activity is specifically required during recovery.

Regulated turnover of protective proteins can determine how long a protective state persists. In rice, OsHDAC1-mediated deacetylation stabilises OsHSA32 by limiting its ubiquitin–proteasome-dependent degradation, providing a mechanistic example of regulated turnover of a heat-protective protein, although its contribution to prolonged memory remains unresolved [70]. Within the proposed model, degradation and autophagy could alter both client availability and regulatory activity during recovery; this interpretation does not imply that protein clearance itself constitutes a general memory storage mechanism.

The same proteostatic process can have different functions at different stages. During the initial stress, enhanced chaperone activity or protein degradation may directly limit damage and improve survival. During recovery, these processes may determine how rapidly damaged proteins are removed, how client pools are redistributed, and how the proteostasis network returns towards its basal configuration. A later alteration in stress responsiveness could therefore arise from changes established during recovery without requiring persistent activation of the pathway itself. Alternatively, sustained chaperone abundance may provide protection during rechallenge and contribute to memory when that functional role is demonstrated. Altered client occupancy could influence the capacity available for the later response, but its causal contribution would require direct testing.

Recent work in *Arabidopsis* has provided evidence that thermopriming involves coordinated regulation of protein maintenance and clearance. Using 90 min of recovery between priming and triggering heat stress, Bajaj et al. [71] showed that priming promotes HSFA1-dependent heat shock responses and activation of the unfolded protein response (UPR), alongside induction of autophagy. Genetic analyses further indicated that the heat shock response (HSR) is essential for survival under the subsequent severe heat treatment, whereas loss of the UPR has a comparatively limited effect on acquired thermotolerance under the conditions tested. The UPR also contributed to subsequent growth recovery. These findings support a central role for the HSR in survival within the broader proteostasis response, while showing that activation of clearance pathways alone is insufficient to ensure survival. Complementary evidence comes from comparisons of total mRNA, polysome-associated mRNA and cotranslational mRNA decay in *Arabidopsis* subjected to severe heat with or without prior priming. Following a two-hour interval between exposures, primed plants retained more polysomes during the severe treatment and restored translational activity more effectively afterwards [72]. These observations show that previous exposure modifies the translational response to the later stress, extending the analysis beyond transcript abundance. These short intervals differ from the recovery periods of several days used to assess prolonged thermomemory involving HSA32, HSFA2/HSFA3 and HSP21–FtsH6 [3,4,5,6]. Neither study establishes whether the same proteostatic requirements sustain protection over longer recovery periods; recovery duration alone, however, does not define a mechanistic boundary between acquired thermotolerance and thermomemory.

### 5.3. Temporal Coupling, Network Remodelling, and Testable Predictions

The proposed state would be defined by both the properties of individual components and their functional relationships. An exposure may alter regulatory competence, proteostatic demand, client handling, and turnover simultaneously, while each property subsequently changes at a different rate. In *Arabidopsis*, manipulation of TARGET OF RAPAMYCIN (TOR) expression altered thermomemory, while glucose–TOR signalling was associated with sustained H3K4me3 enrichment at memory gene promoters after their transcription had returned towards basal levels [73]. A recent *Arabidopsis* dataset combines transcriptomic, proteomic and metabolomic measurements from matched biological material across four days after heat priming, providing a resource for examining coordinated changes during recovery [74]. Such profiles can identify candidate relationships among processes, although causal interactions require experimental perturbation.

To investigate how these regulatory layers contribute to altered responsiveness, comparisons between the initial response and rechallenge could combine matched measurements of chromatin state, transcript abundance, RNA modifications, polysome association and protein modification states. Primed and unprimed plants exposed to the same challenge should be compared at corresponding time points, so that differences attributable to exposure history can be distinguished from differences in treatment severity or sampling time.

A stress-induced change in HSF competence could persist after the initiating signal has declined, while protective components and the processes controlling their turnover remain functionally relevant at different points during recovery. The subsequent response would then depend on how these variables interact when the plant is challenged again.

A similar principle could apply across different stress histories even if the molecular components affected are not identical. Recurrent heat may engage one combination of HSF and proteostatic processes, whereas drought followed by heat may modify other components through partially shared regulatory or proteostatic constraints. Experiments using drought priming followed by heat provide contexts in which to test this possibility but do not yet establish a shared HSF/HSP-dependent mechanism. Conservation across environmental histories should therefore be sought in experimentally demonstrated relationships among processes rather than assumed from the presence of common stress-responsive genes.

The model generates several experimentally testable predictions:

1. At similar HSF/HSP abundance, exposure history may alter HSF promoter occupancy, chaperone–client association and transcriptional reinduction, revealing functional differences not captured by abundance alone.
2. Perturbing protein disaggregation or clearance during recovery should alter aggregate removal kinetics and subsequent stress responses if these processes contribute to memory. Prechallenge damage and physiology should be assessed to distinguish this contribution from unresolved injury.
3. Perturbing the same component during initial exposure, recovery or rechallenge should produce different effects if its function changes across phases. Comparisons with unprimed plants should distinguish memory contributions from acute stress tolerance.
4. Combined manipulation of HSF activity and protein turnover should produce effects on response duration or reinduction that differ from those expected under a predefined model of independent effects if these processes interact functionally.
5. Across species and stress sequences, comparable relationships between HSF activity during recovery, aggregate clearance and subsequent transcriptional reinduction would support conservation of the proposed functional coupling; divergent relationships would define its limits.

For crop resilience, this framework directs attention to whether the duration and resetting of a protective state match the interval between stress episodes. Establishing these relationships could inform strategies to improve responses to recurrent stress, while requiring assessment of growth, reproductive performance, and any costs of maintaining the altered state. Transfer across species or developmental stages cannot be assumed from *Arabidopsis* thermomemory alone.

## 6. Conclusions

Environmental history can alter how plants respond to a subsequent challenge, but a history-dependent phenotype does not by itself identify its molecular basis. The strongest evidence for HSF/HSP-dependent environmental memory comes from recurrent heat, where genetic and mechanistic studies implicate distinct processes in transcriptional regulation, chromatin state, chaperone persistence, protein turnover, and memory resetting. These findings indicate that thermomemory involves contributions from several regulatory and proteostatic processes whose functions and temporal requirements differ.

Whether these relationships extend to broader environmental histories remains unresolved. Drought followed by heat, repeated drought, and other sequential or combined stresses can alter subsequent responses, but the contributions of sustained protection, altered regulatory responsiveness, and incomplete recovery require experimental resolution. Changes in HSFs or HSPs under such conditions identify candidate processes; establishing their involvement in memory requires linking them causally to the effect of previous exposure on the later response.

The framework proposed here extends the focus from persistence of individual stress-associated molecules to the recovery-associated configuration of the HSF/HSP network. Environmental history may alter HSF regulatory competence, chaperone capacity, client handling, protein turnover, and transcriptional competence on different timescales. Functional persistence may therefore involve sustained protective components, changed relationships among processes, or consequences that outlast the initiating molecular change. This concept provides a mechanistic bridge between proteostasis during recovery and later history-dependent responsiveness, while remaining a testable model rather than an established universal mechanism.

The critical experimental advance will be to measure these processes longitudinally across exposure, recovery, rechallenge, and resetting, and to perturb them at defined phases. Extending this approach across stress sequences, tissues, developmental contexts, and species will reveal which contributions are conserved and which depend on the specific environmental history. In crops, determining whether the duration of protection matches recurrent stress patterns, while assessing growth and reproductive performance, will be essential for evaluating its practical relevance.

## Figures and Tables

**Table 1:** Primary evidence for heat memory associated with HSF/HSP.

Component or Process	Experimental Context	Main Finding and Function	Evidence and Limitation	Reference
HSA32	Recurrent heat	Maintains acquired thermotolerance after prolonged recovery.	Genetic; the complete memory state is unresolved.	[3]
HSFA2	Recurrent heat	Extends acquired thermotolerance.	Genetic and physiological; other persistence mechanisms remain unresolved.	[14]
HSA32–HSP101	Heat acclimation	Their interaction prolongs heat memory through posttranscriptional regulation.	Genetic and molecular; the complete network is unresolved.	[15]
HSP21–FtsH6	Priming and recovery	HSP21 supports thermomemory; FtsH6 limits its persistence during recovery and restricts memory duration.	Genetic, protein abundance and physiological analyses; focuses on a plastid proteostasis pathway.	[4]
BRU1	Recurrent heat	Supports sustained expression of memory genes, but is dispensable for initial thermotolerance acquisition.	Genetic and transcriptional; physiological integration remains unresolved.	[17]
HSFA2–HSFA3	Recurrent heat	Heteromeric complexes promote transcriptional memory; genetic analyses support HSFA2/HSFA3 involvement in sustained H3K4me3 enrichment.	Genetic, biochemical and chromatin analyses; direct recruitment of the H3K4 methylation machinery remains unresolved.	[5]
HSFA2–FtsH6	Recovery	HSFA2 activates FtsH6 during recovery, limiting HSP21 persistence and thermomemory duration.	Genetic, promoter-binding and protein analyses; recovery-specific necessity was not isolated.	[6]
Genomic and epigenomic features	Recurrent heat	Memory loci show distinct genomic/epigenomic features and HSFA2/HSFA3 binding patterns, including sustained H3K4me3 enrichment.	Genome-wide binding, expression and chromatin analyses; associations do not establish a causal role for each chromatin feature.	[19]
CDK8–MED12	Recurrent heat	CDK8 supports sustained expression and enhanced reinduction; the tested MED12 allele mainly affects reinduction. HSFA2 recruits CDK8.	Genetic, interaction and transcriptional analyses; phase-specific requirements remain unresolved.	[7]
HSFA2 domains	Heat priming	The oligomerisation domain and other HSFA2 regions contribute to memory competence.	Domain swaps and physiological; relevant interactions remain unresolved.	[8]
Sensory HSF prion-like domains	Heat priming	PrD1 supports chaperone-mediated repression; PrD2 supports heat activation and enhanced transcriptional reinduction after three days of recovery.	Domain deletions, expression, imaging and chromatin-contact assays; acute-response defects limit memory-specific attribution.	[9]
USP1–HSFA2	Repeated heat	USP1 stabilises HSFA2 in vitro; usp1 mutants show impaired heat memory and reduced H3K4me3 enrichment.	Genetic, biochemical and chromatin analyses; the causal link between HSFA2 protection and H3K4me3 remains unresolved.	[26]
HvHSFA2–HvHSFA3	Recurrent heat; barley	Required for barley heat memory, broadly support transcriptional responses to recurrent heat, with no H3K4me3 increase at tested memory loci.	Genetic and physiological; species divergence limits extrapolation.	[28]
NBR1–HSP90.1–ROF1	Priming and recovery	NBR1 mediates HSP90.1/ROF1 turnover; loss of NBR1 enhances heat memory.	Genetic, biochemical and cellular; defines a selective resetting pathway.	[24]

All studies concern *Arabidopsis* unless otherwise indicated. Functions reflect the study findings. Experimental context indicates the treatment or observation period, not necessarily a phase-specific causal requirement. Measurements during recovery and constitutive genetic perturbations do not, by themselves, establish when a factor is required; this requires temporally restricted perturbation. Abbreviations: HSF, heat shock factor; HSP, heat shock protein; H3K4me3, histone H3 lysine 4 trimethylation; Hv, *Hordeum vulgare*; USP1, Universal Stress Protein 1; NBR1, Neighbor OF BRCA1.

**Table 2:** Experimental evidence and unresolved mechanisms of HSF/HSP involvement across environmental histories.

Experimental Situation	Observed Response	HSF/HSP Evidence	Unresolved Question	References
Recurrent heat after recovery	Persistent thermotolerance and transcriptional memory.	Causal contributions of several regulatory and proteostatic processes.	How are these processes coordinated during recovery?	[3,5,7]
Recurrent heat across species	Heat memory can be conserved while downstream regulation diverges.	HvHSFA2 and HvHSFA3 are required for barley heat memory, but their downstream transcriptional regulation differs from *Arabidopsis*.	Which network relationships are conserved across species?	[28]
Dehydration followed by heat; no separate recovery	Pretreatment induces cross stress thermotolerance.	HSP transcript changes accompany the response; persistence after recovery was not tested.	Does protection persist after a defined recovery interval?	[38]
Drought priming followed by drought or heat	Prior drought can improve later tolerance.	HSF/HSP transcripts were assessed after recovery and during subsequent stress in creeping bentgrass; causal involvement remains unresolved.	Do HSF/HSP processes mediate persistence or expression of the response?	[39,40,41,45]
Sequential water deficit and waterlogging	Prior exposure alters physiological responses and recovery.	HSF/HSP involvement in memory has not been established.	Can molecular changes during recovery explain the phenotype?	[46]
Simultaneous heat and salt; integration comparator	HSF regulation supports combined stress tolerance.	HSFA4A integrates stress signalling; the design does not test memory.	Do regulatory changes persist and affect a later challenge?	[47]
Recurrent heat and deficit irrigation	Priming effects depend on genotype and developmental stage.	HSF/HSP involvement in memory has not been established.	Which processes retain the effect of the initial combined exposure?	[48]

Abbreviations: HSF, heat shock factor; HSP, heat shock protein.

**Table 3:** Evidential criteria for establishing HSF/HSP-dependent environmental memory.

Evidence Dimension	Observation or Experimental Requirement	Supported Inference	What Remains Unestablished
Stress responsiveness	HSF activation, HSP induction or another response occurs after exposure.	Activation of a stress response or proteostasis process.	Persistence or environmental memory.
Molecular persistence	A candidate component, modification or molecular state remains altered during recovery.	Persistence after the initial exposure.	Its contribution to the later response.
History-dependent response	Plants with different exposure histories respond differently to the same rechallenge.	Previous exposure modifies subsequent responsiveness.	The process responsible for the difference.
Temporal association	A candidate process changes at times consistent with its proposed function.	A temporal relationship with the phenotype.	Causal involvement.
Functional perturbation	Manipulation changes the history-dependent phenotype; basal physiology and acute tolerance are controlled.	Functional involvement; loss of function may support necessity under the tested conditions.	Sufficiency, direct action or the phase of involvement.
Phase-specific causality	Perturbation restricted to a defined phase changes the later phenotype, with basal and acute response controls.	Causal involvement during the tested phase.	That the process alone constitutes the memory mechanism.
Protection and recovery controls	Protective components and physiological disturbances are assessed before rechallenge; their contributions are tested where feasible.	Clearer attribution to sustained protection, altered responsiveness or unresolved physiological effects.	Complete exclusion of unmeasured contributions.
Resetting	Manipulation changes the duration or disappearance of the history-dependent phenotype.	Active regulation of persistence or resetting.	A universal resetting mechanism.
Proposed model test: coordinated network manipulation	Test whether coordinated manipulation of selected regulatory and proteostatic variables reproduces defined features of the history-dependent phenotype.	If successful, support for the proposed functional relationships among the manipulated variables.	Reconstruction of the complete recovery-associated state, uniqueness of the mechanism, or general applicability.

These criteria are complementary, not a fixed sequence. Interpretation depends on the experimental design, the phase tested and the alternative explanations assessed. The final row describes a proposed experimental strategy for testing the model, not an established mechanism or a prerequisite for demonstrating individual causal contributions. Abbreviations: HSF, heat shock factor; HSP, heat shock protein.

## Data Availability

No new data were created or analyzed in this study. Data sharing is not applicable to this article.
